# Chronic underlying gastrointestinal amyloidosis was revealed by cardiac echography: a case report from Syria

**DOI:** 10.1097/MS9.0000000000001901

**Published:** 2024-03-06

**Authors:** Ali Asaad, Yahia Ranjous, Zein Aldeen Hassan, Nazir Alahmad, Lama Ghanimeh, Ayman Ali

**Affiliations:** a Faculty of Medicine; b Department of Gastroenterology, Faculty of Medicine, Damascus University, Damascus, Syria

**Keywords:** cardiac amyloidosis, gastrointestinal amyloidosis, sparkling appearance, systemic amyloidosis

## Abstract

**Introduction and importance:**

Amyloidosis is an infiltrative disease caused by the deposition of abnormal proteins. While cardiac amyloidosis is relatively common, gastrointestinal (GI) tract involvement is less frequent. In this case, the authors report a delayed diagnosis of systemic amyloidosis presenting mainly with digestive symptoms.

**Case presentation:**

An 81-year-old male presented with the complaint of persistent diarrhoea for over a year and the progressive development of edemas during the last 4 months. Echocardiogram findings revealed the presence of the characteristic sparkling sign. The diagnosis of amyloidosis was confirmed by histopathological biopsies taken from the duodenum. Serum electrophoresis findings strongly suggested the possibility of plasma cell dyscrasia.

**Clinical discussion:**

What distinguishes this case is that the suspicion of amyloidosis as the underlying cause of the diarrhoea did not arise until an incidental echocardiogram revealed cardiac hypertrophy and a sparkling appearance.

**Conclusion:**

This case reminds us to consider amyloidosis as a possible underlying cause for unexplained gastrointestinal symptoms such as diarrhoea, especially in bad economic situations where the diagnosis of rare diseases may be delayed.

## Background

Highlights

- Unique clinical presentation highlights the importance of recognizing atypical symptoms in systemic amyloidosis.
- Diagnostic challenges arise in war zones due to limited access to equipped facilities.
- Delayed medical attention underscores the significance of timely diagnosis in systemic amyloidosis.
- Healthcare providers should not be lenient in requesting investigations, even in challenging circumstances.

Amyloidosis is a collection of rare diseases that involve the deposition and accumulation of misfolded protein in the extracellular space. Depending on the specific protein involved, there are 36 types of amyloidosis, which can be inherited or acquired, systemic or local^1,2^.

Systemic amyloidosis in particular has four main types, light chain amyloidosis (AL) also known as primary amyloidosis which has the maximum gastrointestinal involvement among other types, reactive amyloid amyloidosis (AA) (or secondary amyloidosis), β2-microglobulin amyloidosis (Aβ2M), and transthyretin-related amyloidosis (ATTR)^2–4^.

AL amyloidosis is the most common type of the disease in western world^2^. AL amyloidosis commonly involves heart, kidneys, liver, and notably gastrointestinal (GI) tract, as well as peripheral or autonomic nervous system and other tissues^5^. Recent studies showed that 20% of primary amyloidosis cases are caused by multiple myeloma, a type of cancer affecting plasma cells in the bone marrow, while the remaining cases accompany other plasma cell dyscrasia^2,5^. Further investigations such as bone marrow biopsy with clonal plasma cells greater than or equal to 10%, 24-h urine collection and haematological analysis are necessary for the diagnosis of multiple myeloma^6,7^.

AA amyloidosis is mainly associated with chronic inflammatory conditions, making it the leading cause of systemic amyloidosis in developing countries, unlike other systemic forms AA amyloidosis rarely affects the heart^2,8^.

Hence systemic amyloidosis is a multiorgan disease, some common symptoms include diarrhoea, GI tract bleeding, dyspnoea due to constrictive heart failure, general weakness, peripheral neuropathy, and more^3^. Seventy percent of patients have renal symptoms at presentation, making it the most frequent manifestations of systemic amyloidosis^9^.

Diagnosing systemic amyloidosis is challenging because the clinical symptoms associated with different types of the disease are similar^10^. Also, most patients present with asthenia and dyspnoea, which are not specific symptoms and may lead to delayed diagnosis^9^.

In cardiac amyloidosis, a granular sparkling appearance on echocardiography is characteristic but has 87% sensitivity^11–13^. While a suggestive clinical presentation is important for diagnosing systemic amyloidosis, the definitive confirmation requires a tissue biopsy with Congo red stain or apple green birefringence, an investigation that requires adequately equipped facilities that are not always available in war times^14^.

In war times and crisis situations, when investigations are not performed as recommended in the guidelines due to lack of recourse and patients’ noncompliance, doctors must focus on all symptoms, even those that are tolerated by the patient. Moreover, they should spend more time in informing the patients about their disease and emphasize the significance of regular follow-up appointments in ensuring the effectiveness of treatment. In our case, the patient endured chronic diarrhoea without seeking medical attention until the onset of oedema and dyspnoea. The suspicion of amyloidosis arose when a sparkling appearance was detected during the cardiac echocardiography, prompting the subsequent performance of an endoscopy.

In this case, we report a delayed diagnosis of systemic amyloidosis presenting mainly with digestive symptoms. The suspicion of amyloid disease was only aroused after the echocardiogram showed cardiac hypertrophy and sparkling sign. This case report has been written in line with the SCARE guideline^15^.

## Case presentation

An 81-year-old male presented with the complaint of persistent diarrhoea and the progressive development of edemas. He experienced diarrhoea episodes occurring 3–4 times a day, which started ~1 year ago. The diarrhoea was accompanied by non-specific generalized abdominal pain. The symptoms did not wake him up from sleep and were not alleviated by fasting. Over the course of the past four months, the patient developed a progressive oedema in the lower extremities accompanied by dyspnoea and a decline in urine output. The patient did not have a relevant medical history. The clinical examination unveiled decreased breath sounds at the lung bases and second-grade oedema in the lower extremities. Laboratory tests indicated renal insufficiency (creatinine: 2 mg/dl), decreased serum albumin (serum albumin: 2.2 g/dl), respiratory alkalosis (pH: 7.45, PaCo2: 35 mmHg), and normocytic anaemia (Hb: 12 g/dl, MCV: 96 fl). Chest radiography revealed the presence of bilateral effusion, which was confirmed to be of a transudate nature through thoracentesis. Abdominal echography revealed congestion in the suprahepatic veins. Echocardiogram findings revealed cardiac hypertrophy (left ventricle thickness: 16 mm, right ventricle thickness: 9 mm, left atrium diameter: 4.7 cm, ejection fracture: 45%), the onset of restrictive cardiomyopathy, and the presence of the sparkling sign, which is characteristic for the amyloid disease. Computed tomography (CT) scan results demonstrated bowel wall thickening. These aforementioned findings raised a strong suspicion of amyloidosis. Esophagogastroduodenoscopy macroscopic findings were within normal, and biopsies were taken to confirm the diagnosis. Pathological examination confirmed the presence of amyloid deposits using congo red stain. Serum protein electrophoresis was performed to identify the underlying cause of the amyloidosis. The test revealed a decrease in alpha 1 globulin and albumin levels, as well as an increase in gamma globulin. These aforementioned findings strongly suggested the possibility of plasma cell dyscrasia. Unfortunately, because of the economic crisis and the patient’s advanced age, he declined to undergo further haematological investigations to confirm his condition. Instead, based on the patient’s will, he was discharged on palliative treatment for the diarrhoea and edemas.

## Discussion

Amyloidosis is an infiltrative disease caused by the deposition of abnormal proteins. This condition manifests as localized amyloidosis, affecting one organ, or systemic amyloidosis, affecting multiple organ^3^. The most prevalent form of systemic amyloidosis is light chain amyloidosis, wherein the accumulated protein is the light chain (kappa or lambda) of the immunoglobulin produced by plasma B cells^1^.

The involvement of the GI tract in amyloidosis is relatively uncommon, but has a bad effect on the general prognosis^16,17^. Symptoms can vary depending on the location and extent of amyloid deposits. GI bleeding, malabsorption, and protein-losing gastroenteropathy are some of the most important clinical manifestations in GI amyloidosis^18^. Conversely, cardiac amyloidosis is more common and typically presents with non-specific symptoms associated with heart failure, such as fatigue and dyspnoea^19^. Early detection of cardiac amyloidosis is important, as it is the main determinant of prognosis and survival rates^20^. Various investigations can be performed in the work-up to diagnose cardiac amyloidosis, including electrocardiogram, echocardiogram, MRI, and biopsy^19^. Although not specific, the sparkling appearance observed on echocardiography was the initial indication that led us to suspect amyloidosis in our patient.

Diagnosing amyloidosis can be challenging due to the wide range of non-specific symptoms, which vary depending on the affected organ. Additionally, amyloidosis primarily affects elderly patients, who are more predisposed to other more common diseases^21^. Therefore, it is not uncommon for the diagnosis to be delayed by over a year, typically requiring consultations with multiple physicians before reaching a definitive diagnosis^22^. This issue is particularly prevalent during times of crisis and bad economical situations. For example, physicians may skip essential investigations and instead start with symptomatic treatment, aiming to alleviate the financial burden on the patient. Furthermore, the patient may struggle to maintain regular follow-ups with doctors due to bad financial situation and the difficulty of reaching public hospitals. Moreover, patients often postpone seeking medical help, hoping for spontaneous recovery.

The elevation in gamma globulin levels and the presence of cardiac involvement suggest that our patient has light chain amyloidosis (AL amyloidosis), which is associated with plasma cell dyscrasias. Unfortunately, the patient refused to continue the haematological investigations and decided to pursue palliative treatments instead.

Our case represents a delayed diagnosis of systemic amyloidosis with cardiac involvement, initially presenting as chronic diarrhoea persisting for over a year. Notably, what sets this case apart is that the suspicion of amyloidosis as the underlying cause of the diarrhoea did not arise until an incidental echocardiogram revealed cardiac hypertrophy and a sparkling appearance. Unfortunately, this delayed diagnosis of amyloidosis may have contributed to a potentially worsened prognosis.

## Conclusions

This case reminds us to consider amyloidosis as a possible underlying cause for unexplained gastrointestinal symptoms, such as diarrhoea. It also emphasizes the significance of ensuring that patients are well-informed about the necessity of timely follow-up if the symptoms persisted. This becomes particularly important during times of war when accessing healthcare services may pose challenges.

## Ethical approval

Not applicable.

## Consent

Written informed consent was obtained from the patient for publication of this case report and any accompanying images.

## Source of funding

The authors did not receive funding for this paper.

## Author contribution

All authors took part in writing the manuscript. All authors read and approved the final manuscript.

## Conflicts of interest disclosure

Not applicable.

## Research registration unique identifying number (UIN)

Not applicable.

## Guarantor

Yahia Ranjous.

## Data availability statement

The laboratory tests and imaging results are available from the corresponding author on reasonable request.

## Provenance and peer review

The paper was not invited. Not commissioned, externally peer-reviewed.
